# Pathways for external alkalinization in intact and in microwounded Chara cells are differentially sensitive to wortmannin

**DOI:** 10.1080/15592324.2017.1362518

**Published:** 2017-08-14

**Authors:** Alexander A. Bulychev, Ilse Foissner

**Affiliations:** a Department of Biophysics, Faculty of Biology, Moscow State University, Moscow, Russia; b Department of Cell Biology and Physiology, Division of Plant Physiology, University of Salzburg, Salzburg, Austria

**Keywords:** Characean algae, confocal fluorescence microscopy, pH microelectrodes, alkaline cell regions, nonuniform photosynthetic activity, microwounding

## Abstract

Proton flows across the plant cell membranes play a major role in electrogenesis and regulation of photosynthesis and ion balance. The profiles of external pH along the illuminated internodal cells of characean algae consist of alternating high- and low-pH zones that are spatially coordinated with the distribution of photosynthetic activity of chloroplasts underlying these zones. The results based on confocal laser scanning fluorescence microscopy, pH microsensors, and pulse-amplitude-modulated chlorophyll microfluorometry revealed that the coordination of H^+^ transport and photosynthesis is disrupted by the 2 different environmental cues (low light and wounding) and by a chemical, wortmannin interfering with the inositol phospholipid metabolism. On the one hand, the transition from moderate to low irradiance diminished the peaks in the profiles of photosystem II (PSII) quantum efficiency but did not remove the pH bands. On the other hand, the microwounding of the internode with a glass micropipette, impacting primarily the cell wall, resulted in a rapid local alkalinization of the external medium (by 2–2.5 pH units) near the cell surface, thus mimicking the appearance of natural pH bands. Despite their seeming similarity, the alkaline bands of intact cells were eliminated by wortmannin, whereas the wound-induced alkalinization was insensitive to this drug. Furthermore, the attenuation of natural pH bands in wortmannin-treated cells was accompanied by the enhancement in spatial heterogeneity of PSII efficiency and electron transport rates, which indicates the complexity of chloroplast–plasma membrane interactions. The results suggest that the light- and wound-induced alkaline areas on the cell surface are associated with different ion-transport systems.

**Abbreviations**
pH_o_: pH of outer medium near cell surface
LL: local light (localized illumination)
PFD: photon flux density
PM: plasma membrane
PSII: photosystem II
Wmn: wortmannin
Y(II): effective quantum yield of electron transport in PSII

## Introduction

Characean algae exposed to light generate alternating acid and alkaline bands in the external unstirred layers along their internodal cells,^1,2^ with pH varying from 6.5 to 10.0 at a mm-length scale.^3^ This ability is essential for photosynthesis of algae inhabiting weakly alkaline waters, because acid zones facilitate the supply of inorganic carbon across the plasma membrane (PM) by converting a charged species HCO_3_^−^ prevailing in the environment into a neutral membrane-permeable form, CO_2_^4^ The high pH areas are also indispensable, since the passive H^+^ influx (or OH^−^ efflux) in these regions counterbalances the positive charge transferred by the PM H^+^-ATPase in the acid zones, thus ensuring steady-state electrogenesis and pH homeostasis. The pH pattern on the cell surface is mirrored by uneven distribution of photosynthetic activity over the cell length.^5,6^ The alkaline peaks in the longitudinal pH profiles coincide with the cell areas where photosynthesis is inhibited because of the low availability of inorganic carbon. Conversely, the acid bands are collocated with the cell regions where photosynthetic rates are high.

The cytoplasm under the acid zones in *Chara* cells contains numerous PM invaginations enriched with the H^+^-ATPase, termed charasomes that enlarge the area of H^+^ efflux, thus promoting availability of CO_2_^7,8^ Charasomes are long-lived light-dependent structures; they disappear after 7–10 d of darkening. The elongation of characean internodes is largely limited to cell regions under the acid bands,^9^ in consistency with the acid-growth hypothesis and with the higher photosynthetic activity in these regions. Cortical mitochondria accumulate in photosynthetically active cell regions under the acid bands and are depleted in other cell parts.^10^

The alkaline bands could be induced under light in targeted cell regions, provided the chloroplasts were mechanically removed from these regions by rubbing a turgorless cell with a thread.^11^ Foissner et al.^8^ described later that the alkalinization at wounding-induced chloroplast-free regions is due to the presence of an uneven wound wall. The wound-elicited alkaline zones looked similar to the usual light-dependent pH bands. The apparent similarity does not yet prove the identity of mechanisms for light-dependent formation of high pH zones in intact and wounded cells. The mechanical detachment of chloroplasts is a severe injuring treatment, after which only few cells survived. Microperforation of the cell wall by a glass microneedle exerts minimal injury and can be made repeatedly in multiple places of the same cell.^12^ After cell wall incision the external pH at the point of wounding increased rapidly, approaching to 9.5–9.7 that is close to pH_o_ at the electrochemical equilibrium for protons across the PM. The external alkalinization at the microperforation site was detected thus far with pH microelectrodes; the demonstration of this phenomenon by independent techniques with a precise spatial resolution is still awaited. It is not yet known whether different environmental cues – photosynthetically active light and the mechanical stress – mobilize identical or distinct transport systems in the PM. Eliciting the action potential, associated with a hundredfold increase in cytosolic Ca^2+^ level, arrested the H^+^ fluxes in illuminated cells and diminished the pH band formation, whereas it enhanced the incision-induced H^+^ flow.^12^ However, the effects of metabolic inhibitors on flows elicited by different stimuli have not been compared.

A recent study revealed that the light-induced pH bands in *Chara* cells disappear after the treatment with an inhibitor of phosphoinositide 3-kinases (PI-3 kinase), wortmannin,^13^ indicating the involvement of phosphorylated phosphoinositides in the generation of pH bands. Phosphoinositides have an important regulatory role in cell physiology. The appearance of tubular structures reminiscent of charasomes after the inhibition of lipid phosphorylation by wortmannin indirectly suggests the rearrangement of lipid material trafficking and possible disturbance of the charasome-mediated proton extrusion.^13^ Since the pH banding pattern in resting cells is coordinated with the patterns of photosynthetic activity and charasome frequency, the influence of wortmannin on photosynthetic activity is likely.

Remarkably, morphological changes during wound healing were not appreciably affected by wortmannin. Considering that the rise of external pH near the cell surface is among the early events in wound healing, it is important to find out whether the pH increase in response to microperforation of the cell wall is as sensitive to wortmannin as the light-induced pH bands.

In this study we examined the influence of the PtdIns-3 kinase inhibitor wortmannin on light-dependent profiles of cell surface pH (pH_o_) and photosystem II (PSII) activity, as well as on the local increase in surface pH after cell wall microperforation. The pH indicator phenol red is inconvenient for the detection of local pH changes on a 10- to 100 µm scale because of the interference by background absorbance of this dye distributed over a thick water layer. We applied fluorescence and confocal laser scanning microscopy to visualize the pH differences at the cell surface with the aid of fluorescein isothiocyanate (FITC)-dextran. This method is better suited for detection of small pH spots than the use of phenol red. Quantitative measurements were made with pH microelectrodes allowing spatial resolution ∼10 µm. Unlike phenol red whose color transition is limited to the pH range 6.8–8.2, pH microelectrodes show a linear response in the pH range 2–10.^14^

## Results

### Influence of light intensity and wortmannin on profiles of surface pH and PSII activity

**Figure 1.**
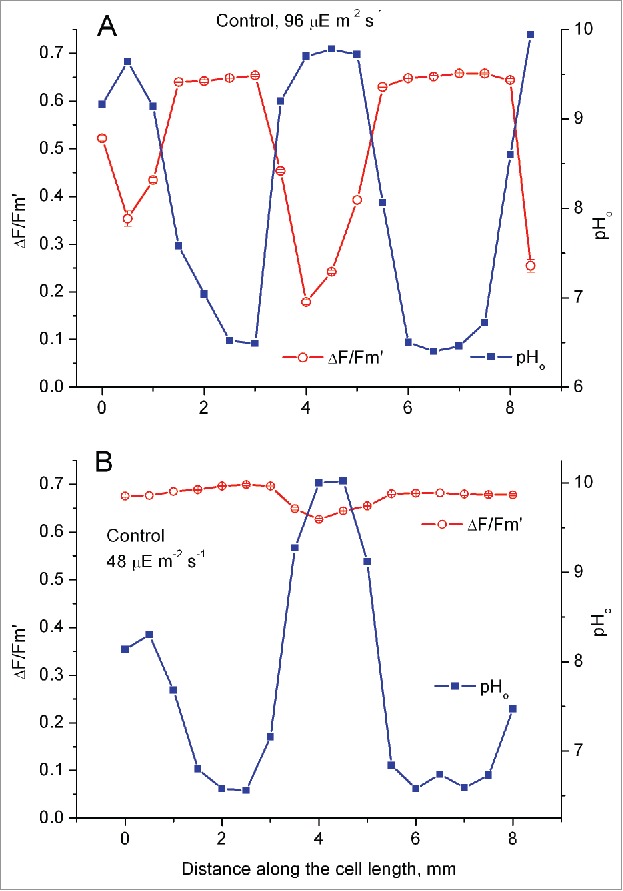
Longitudinal profiles of the effective PSII quantum yield (Δ*F*/*F*_m_') and the local pH on the cell surface (pH_o_) in a *Chara* branchlet internode at photon flux densities of 96 µmol m^−2^ s^−1^ (A) and 48 µmol m^−2^ s^−1^ (B).

Figure 1 shows the inverse relationship between the pH near cell surface (pH_o_) and the effective quantum efficiency of PSII Y(II) in longitudinal profiles that were measured on a branchlet internodal cell at elevated and reduced light intensities. The spatial variations of the effective PSII quantum yield were remarkably large at elevated irradiance (96 µmol m^−2^ s^−1^). At some locations under the alkaline band the parameter Y(II) dropped below 0.2; by contrast, Y(II) was rather high (∼0.65) at low pH_o_ regions (Fig. 1A). Accordingly, the relative rates of linear photosynthetic electron transport under the alkaline band were nearly 3.5 times lower compared with acid bands. At PFD of 48 µmol m^−2^ s^−1^ (Fig. 1B), peaks in the Y(II) profile were diminished but the inverse relation between pH_o_ and Y(II) was still evident. The reduction in light intensity suppressed some alkaline bands but the main peak remained undiminished. Similar reduction in the number of alkaline peaks under dim light is also characteristic of the main axis internodes.^15^

**Figure 2.**
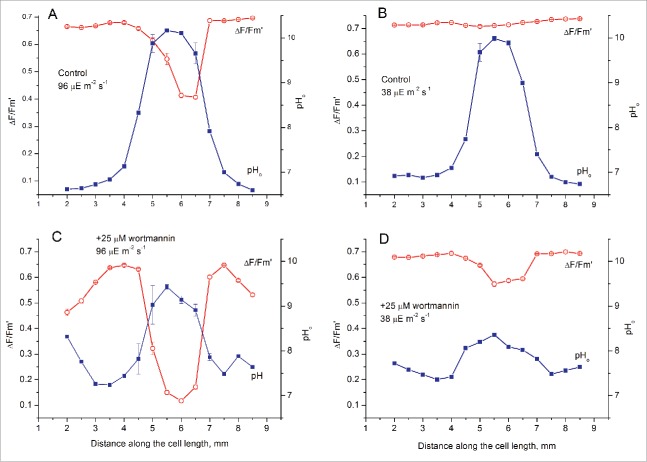
Longitudinal profiles of the effective PSII quantum yield (Δ*F*/*F*_m_') and the local pH on the cell surface (pH_o_) in a *Chara* branchlet internode in the absence (A, B) and presence (C, D) of wortmannin at photon flux densities of 96 µmol m^−2^ s^−1^ (A, C) and 38 µmol m^−2^ s^−1^ (B, D). The cell was incubated in the presence of 25 µM wortmannin for 2 h before measurements.

Figure 2 displays the longitudinal profiles of pH_o_ and the effective quantum yield of PSII Y(II) that were measured in the absence and presence of 25 µM wortmannin (Wmn) at 2 PFD levels (96 and 38 µmol m^−2^ s^−1^). In the absence of the inhibitor, the pH_o_ profiles showed large pH variations at both PFD levels (Fig. 2A, B). At elevated PFD the inverse relation between the pH on the cell surface and the effective quantum yield of PSII activity was apparent, while at reduced PFD the Y(II) profile was almost flattened. The loss of correlation between Y(II) and pH_o_ at lowered photon flux densities indicates that the photosynthetic electron flow is constrained by CO_2_ availability at light sufficiency but this constraint is released under light-limiting conditions.

The incubation of the cell for 1.5 h in the presence of 25 µM Wmn reduced the amplitude of pH peaks. At both light intensities, the pH_o_ shifted upward in the acid zones and downward in the alkaline zone. These changes point to the inhibition of circular electric currents flowing in a circuit comprising the plasma–membrane H^+^-ATPase and the “high pH channels.” The pH profile was suppressed by Wmn stronger at the reduced PFD (38 µmol m^−2^ s^−1^) than at the elevated PFD (Fig. 2C, D). The incubation with Wmn was accompanied by inhibition of PSII activity, primarily under the alkaline band (Fig. 2C, D). The inhibition of PSII activity under the alkaline bands was milder under low light intensity (Fig. 2C, D). Because Wmn differentially inhibited PSII activity under the acid and alkaline bands, the spatial heterogeneity of the PSII quantum yield was enhanced at both light intensities (cf. Fig. 2A vs. 2C and Fig. 2B vs. 2D). Thus, the longitudinal pH profiles were smoothed under the action of Wmn, whereas the nonuniform distribution of PSII efficiency increased. This type alteration in the patterns of pH_o_ and Y(II) was previously observed after the action potential generation in *Chara* cells.^6^

**Figure 3.**
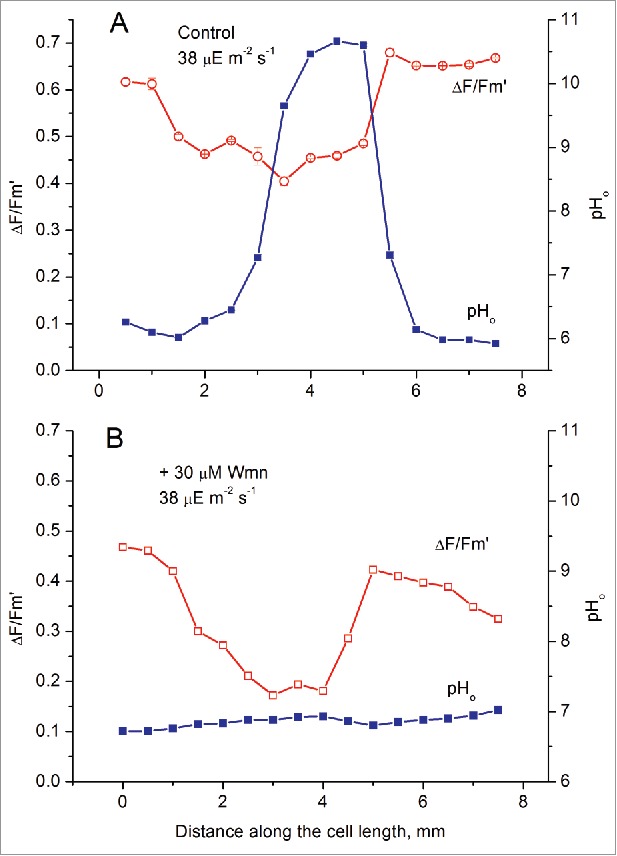
Elimination of pH bands accompanied by nonuniform distribution of the effective quantum yield of PSII photoreaction (Δ*F/F*_m_') after the treatment of the main axis internode with 30 µM wortmannin. (A) Control conditions; (B) after 1-h incubation in the presence of wortmannin.

After incubation of the cell at elevated concentrations of the inhibitor (30 and 50 µM) the longitudinal pH profile was flattened entirely (Fig. 3). Figure 3B shows the nearly uniform pH_o_ profile at PFD of 38 µmol m^−2^ s^−1^. A similar flattened pH profile was also measured at 96 µmol m^−2^ s^−1^ (data not shown). At these concentrations of Wmn, strong depression of PSII quantum yield occurred not only under the alkaline zones but also under the acid zones. Nevertheless, the spatial heterogeneity of Y(II) still existed despite the uniform distribution of pH_o_. Unlike the disrupted coordination between Y(II) and pH_o_ under dim light, which was due to flattening of the Y(II) profile (Fig. 2B), the disruption in Wmn-treated cells was caused by flattening of the pH_o_ profile (Fig. 3B). This loss of coordination between Y(II) and pH_o_ indicates that the pattern of photosynthetic activity in the chloroplast layer was more resistant to the inhibitory treatment than the pattern of H^+^ fluxes across the PM. It appears that the heterogeneity at the chloroplast layer can persist after eliminating the nonuniform profile of the surface pH and that spatial variations of photosynthetic activity are not only due to differential uptake of CO_2_.

**Figure 4.**
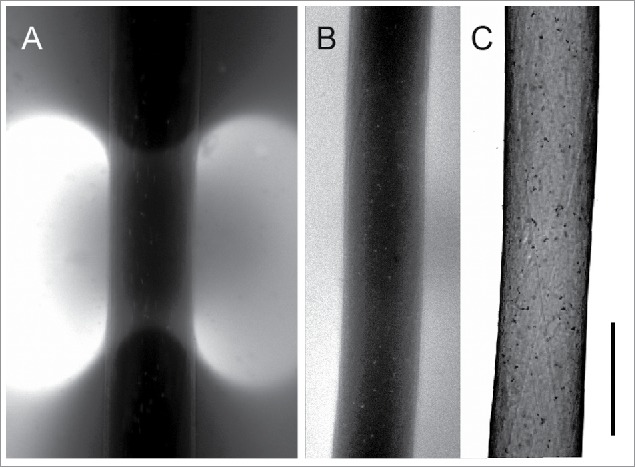
Elimination of a pH band visualized by fluorescent FITC-dextran after treatment of a branchlet internodal cell with 50 µM wortmannin. (A) Alkaline band formed under a light intensity of about 90 µmol m^−2^ s^−1^ for 15 min in artificial pond water; the brightest fluorescence corresponds to a pH ≥ 7.5, the lowest fluorescence corresponds to a pH ≤ 6; (B) homogeneous pH along the cell surface after 2 hours treatment with wortmannin; (C) corresponding bright field image. Bar is 500 µm.

The effect of Wmn on the pH banding pattern was visualized by FITC, a pH-indicating dye (Fig. 4). For our study we used FITC coupled to dextran with a molecular weight of 70 kDa. This conjugate is too large to penetrate the cell wall and cross the plasma membrane and is therefore suitable to monitor changes in extracellular pH. Fig. 4A shows a brightly fluorescent alkaline band flanked by low fluorescent acid regions. After 2-hour treatment with Wmn the pH along the cell surface became uniform (Fig. 4B; Fig. 4C is the bright field image of the cell).

### Cyclosis-mediated transmission of a photosynthetically active metabolite

**Figure 5.**
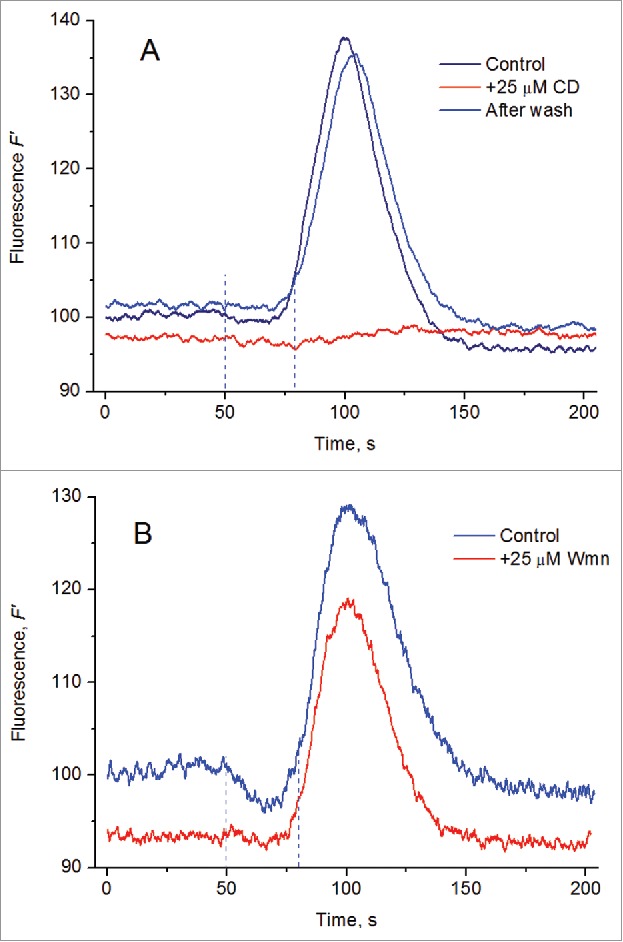
Cyclosis-mediated changes of *F'* chlorophyll fluorescence elicited by local illumination of a distant cell region. (A) Suppression of *F'* changes after the arrest of cyclosis with 25 µM cytochalasin D and its reversal upon washing the cell with fresh medium; the optic fiber tip was at a 1.5-mm distance from the point of fluorescence measurements; each curve is an average for *n* = 4. (B) Chlorophyll fluorescence *F*′ transients observed at a 1-mm distance from the site of local light (LL) application in untreated cells and the cells treated for 1 h with 25 μM wortmannin. Curves are average traces obtained from 8 and 10 records for untreated and wortmannin-treated cells, respectively. Duration of LL pulse was 30 s; background light intensity, 8 μmol m^−2^ s^−1^. Vertical dashed lines mark the period of LL action. Fluorescence curves are normalized to the *F'* level in untreated cells before local illumination.

Photosystem II in characean internodes is subject to long-distance photoregulation. When a small portion of the internodal cell is shortly exposed to local illumination, a transient increase in actual chlorophyll fluorescence (*F'*) can be observed after a lag period on mm-scale distances, provided the analyzed cell area is positioned downstream of the illuminated region.^16, 17^ This fluorescence transient is mediated by cytoplasmic streaming. It vanishes after the cessation of cyclosis in the presence of 25 μM cytochalasin D and reappears after the recovery of cyclosis in washed cells (Fig. 5A). Its origin presumably comprises: (1) the export of excess reducing equivalents and primary assimilates from the chloroplasts in the area of bright local illumination, (2) the downstream transfer of these substances with the cytoplasmic flow, (3) the import of delivered reductants by chloroplasts in shaded cell regions, and (4) the eventual reduction of plastoquinone and the primary quinone Q_A_. When the distance between the local light source and the analyzed area was comparatively short (1 mm), the transient increase in actual fluorescence was often preceded by a small decrease in fluorescence (Fig. 5B, upper curve). The addition of 25 µM Wmn had no appreciable influence on the cyclosis-mediated increase in *F'* fluorescence but eliminated the initial drop of *F'* fluorescence (lower curve). The appearance of this component in the fluorescence response was tentatively assigned to light-activated circular electric currents between the acid and alkaline domains.^17^ The selective inhibition of this component in Wmn-treated cells is in accord with the suppression of pH bands and the underlying circular currents. At the same time, the transfer of reducing equivalents with the cytoplasmic flow, accounting for the peak of *F’* fluorescence, was undisturbed in the presence of Wmn.

### Induction of alkaline spots after microperforation of the cell wall

**Figure 6.**
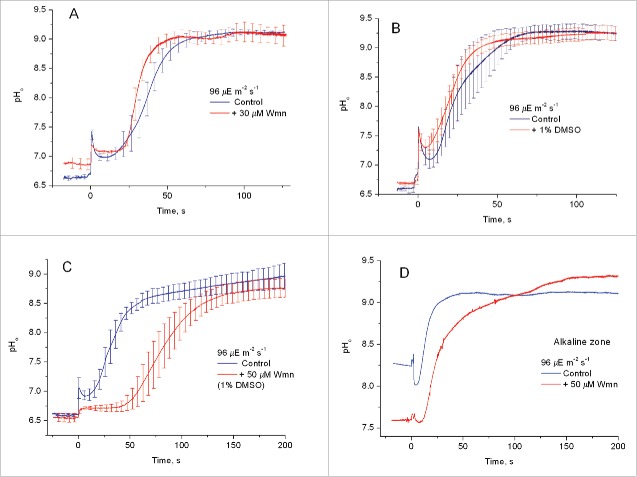
Formation of the external alkaline spot at the site of cell wall microperforation in the absence and presence of wortmannin. (A) Changes in pH_o_ induced by the microwounding before and after a 2-h treatment with 30 μM wortmannin (0.3% DMSO); (B) the absence of side effect of 1% DMSO on pH changes induced by microperforation; (C) the pH_o_ response to microwounding before and after a 2-h incubation of the internode with 50 μM wortmannin (1% DMSO); (D) the pH_o_ response to microwounding in a slightly alkaline zone before and after the wortmannin treatment specified in (C). Plots in (A–C) are averaged records ± SE for 3–8 replicates made in different cell sites. Curves in (D) are representative records. Zero time corresponds to the moment of microperforation.

Figure 6 shows the local pH_o_ changes induced by microperforation of the internodal cell with a glass microneedle having a tip diameter ∼1 µm. The treatment was applied as a short insertion into the cell wall or the cytoplasm followed by an immediate withdrawal of the microneedle. It is seen in Fig. 6A that 2-h incubation in the presence of Wmn diluted to 30 µM from a 10 mM stock solution had no significant influence on the pH response to cell wall microperforation at PFD of 96 µmol m^−2^ s^−1^. Also at PFD of 38 µmol m^−2^ s^−1^, the amplitude and kinetics of the incision-induced pH changes in Wmn-treated cells showed no significant difference from those in untreated cells (data not shown).

The addition to the medium of 1% DMSO had no significant influence on the pH rise induced by microperforation (Fig. 6B). However, when Wmn was diluted 100-fold from a 5 mM stock solution in DMSO, the incision-induced pH rise was retarded (Fig. 6C). This delay observed in the presence of 50 µM Wmn cannot be ascribed to the osmotic effect of the solvent because DMSO is slightly permeable across the biologic membranes (the reflection coefficient of the PM σ = 0.73–0.85).^18^ According to Shimmen and Ogata^19^ the difference in DMSO concentration between the intracellular and external medium disappears within 3 h after the addition of 200 mM DMSO to the outer solution. Since our measurements lasted for several hours after preliminary 2-h incubation in DMSO-containing medium, the internal hydrostatic pressure was largely equilibrated in these experiments and cannot account for the delayed pH rise.

Microperforation of the cell in a weakly alkaline area (Fig. 6D) induced a smaller pH_o_ shift because of the initially higher pH value and a fixed upper limit of pH_o_. Following the Wmn treatment, the pH in the formerly alkaline zone was lowered, reflecting the smoothing of the pH_o_ profile, while the amplitude of wound-induced pH changes increased accordingly. The initial shift of the electrode potential in the alkaline zone at the instant of perforation was opposite to that in the acid area. This shift seems to arise from the extracellular recording of circular electric currents flowing in opposite directions in the acid and alkaline zones. Data in Fig. 6 prove that Wmn had no inhibitory action on the pH increase induced by cell wall microperforation.

**Figure 7.**
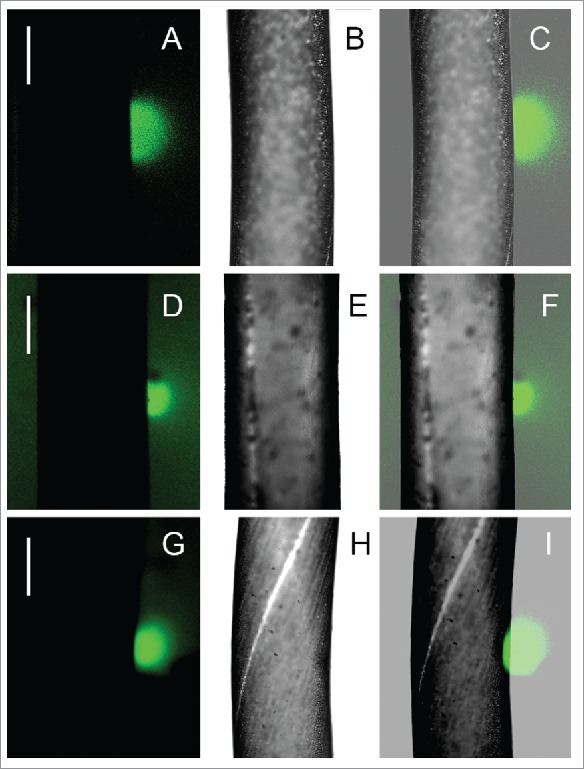
Formation of external alkaline zones, visualized by fluorescent FITC-dextran at the point of cell wall microperforation in the absence and presence of wortmannin. Alkaline spot at a perforated region of an untreated cell (A–C); of a cell treated with 0.5% DMSO for 2 hours (D–F); and of a cell treated with 50 µM wortmannin for 2 hours (G–I). A, D, and G are the fluorescence images; B, E and H are the corresponding bright field images; C, F and I are the merged images. Bars are 200 µm.

Figure 7 shows the FITC-dextran fluorescence at the surface of branchlet internodal cells that were microperforated in acidic areas. Patterns were similar when the cells were injured in artificial pond water (Fig. 7A-C), in solvent-containing artificial pond water (Fig. 7D-F) or in artificial pond water supplemented with Wmn (Fig. 7G-I). Bright fluorescence around the perforation site was confined to a narrow spot whose surface area was approximately 2 orders of magnitude lower than that of conventional pH bands (compare Figs. 4 & **7**).

## Discussion

The results revealed that wortmannin effectively inhibits and fully eliminates the light-dependent pH bands in branchlet and main axis internodes of *Chara* cells without inhibiting the formation of high pH at the site of microwounding. This differential sensitivity of alkaline zones induced in intact and microwounded cells suggests that these zones result from either activation of different transporters or from operation of different regulatory pathways targeted on identical transporters. We favor the first alternative because the inactivation of high pH channels, manifested as an abrupt increase in membrane resistance concurrent with the decline of alkaline pH_o_ after eliciting the action potential,^20^ did not inactivate the transporters responsible for the alkaline shift at the site of microperforation. Moreover, the pH response to microwounding was stimulated after the cell excitation.^21^

Simultaneous measurements of pH_o_ and the PSII quantum efficiency revealed that the coordination of pH_o_ and Y(II) profiles disappears under low light intensity in untreated cells and at elevated irradiance after the treatment with wortmannin. At low PFD the effective quantum yield was insensitive to variations of local pH on the cell surface (Fig. 2B). This indicates that the structural differences, such as abundance of charasomes under the acid zones and scarcity of these organelles under the alkaline zones have no influence on photosynthetic activity at light-limiting conditions, when the photon flux rather than CO_2_ availability become the rate-limiting factor of photosynthesis.

On the other hand, the wortmannin treatment suppressed spatial variations in the pH_o_ profile with a concomitant enhancement in spatial variations of Y(II). The suppression of plasma-membrane H^+^-ATPase and H^+^(OH^−^) conducting channels might indicate continuous recycling of the respective PM proteins, which is inhibited by Wmn, an inhibitor of intracellular vesicle transport.^13^ It is possible that the recruitment of the transporters to the PM occurs via a PtdIns-3 kinase-dependent pathway.

The light-dependent distribution of pH_o_ along the cell length was smoothed or eliminated by Wmn, whereas the distribution of Y(II) was enhanced and became even more contrast. Similar modifications of pH_o_ and Y(II) profiles were also observed after eliciting the action potential.^6^ Since the mechanism of excitation in *Chara* involves phosphoinositides,^22^ the aforementioned similarity might have a common origin, thus far hidden. The action potential is associated with a large (∼100-fold) increase in cytosolic Ca^2+^ level, which may affect the chloroplast function through the Ca^2+^ uniport system of the chloroplast envelope and owing to the Ca^2+^-sensitivity of the stromal enzymes involved in the carbon reduction cycle.^23,24^ Remarkably, the Wmn treatment elevated the cytoplasmic Ca^2+^ content in *Medicago truncatula* root hairs and disturbed the Ca^2+^ signaling in *Arabidopsis*.^25,26^ The impairment of Ca^2+^ homeostasis by Wmn, acting primarily on intracellular vesicular transport and metabolism of phosphoinositides, seems possible. The assumed elevation of cytoplasmic Ca^2+^ after the Wmn treatment would be consistent with the lack of the inhibitory effect of Wmn on alkaline zones produced by microperforation. On the other hand, cytoplasmic streaming, a prerequisite for pH banding, continues at near control rates in cells treated with Wmn,^13^ which argues against a considerable elevation of cytoplasmic free Ca^2+^.

In summary, wortmannin was shown to be a specific inhibitor of light-dependent pH banding, exerting no inhibitory action on the wound-induced pH spots. This dissimilarity points to the existence of different transport systems accounting for proton or hydroxyl flows in intact and microwounded cells. Thus, wortmannin might represent a useful tool for discriminating the cellular events associated with these distinct H^+^/OH^−^-transporting systems.

## Materials and methods

### Plant material

*Chara corallina* algae were grown in glass vessels at room temperature under scattered daylight (photon flux density ∼10 µmol m^−2^ s^−1^ during daytime). Branchlet internodal cells and the main axis internodes measuring ∼1 cm and 4–8 cm in length, respectively, were used and yielded similar results. Isolated internodal cells were placed into artificial pond water containing 0.1 mM KCl, 1.0 mM NaCl, and 0.1 mM CaCl_2_. The pH of the medium was adjusted to pH 7.0 with NaHCO_3_. After cutting the cells from the string, they were allowed to stay in the medium for at least 1 day. Isolated cells were mounted horizontally in a transparent chamber with a volume of 5 or 40 mL, depending on cell size, and placed on a stage of an inverted fluorescence microscope, Axiovert 25-CFL (Zeiss, Germany). Microscopic observations and fluorescence measurements were made at 23°C on the chloroplast layer in the lower cell side. The chloroplasts in characean internodes are immobile; they are aligned in rows extending along the streamlines of fluid flow. The velocity of cytoplasmic streaming was 60–90 µm/s.

### Measurements of modulated fluorescence

Parameters of chlorophyll fluorescence in vivo were measured on microscopic cell regions (diameter ∼100 µm) using a Microscopy-PAM fluorometer (Walz, Germany) equipped with a × 32/0.4 objective lens. The excitation/emission light beams used for microfluorometry, the position of an optic fiber used for local photostimulation of a distant cell area, and the background illumination of the whole internode were arranged as shown schematically in a previous work.^27^ The actinic illumination of the whole cell was provided from a microscope upper light source through a 5-mm-thick blue glass filter SZS-22 (λ < 580 nm). The intensity of actinic illumination was attenuated by neutral density glass filters. Data in graphs represent the effective quantum yield of PSII operation calculated from the maximal fluorescence *F*_m_' attained during saturating light pulses and the actual fluorescence *F’* in actinic light: Y(II) ≡ Δ*F*/*F*_m_’ = (*F*_m_’ – *F*’)/*F*_m_’. The signal from a photomultiplier was fed into the Control Unit of the pulse-amplitude modulation system and processed with WinControl-3 software. In addition, the signal was digitized by means of an AD converter PCI-6024E (National Instruments, USA), and displayed on a computer monitor using WinWCP program (Strathclyde Electrophysiology Software). Data points were sampled at regular intervals of about 51 ms.

### Measurements of surface pH

The local pH in the outer medium (pH_o_) was measured at a distance of about 10 µm from the cell surface with glass-insulated antimony pH-microelectrodes having tip diameters of 3–15 µm. The slope of the electrode function was approximately 53 mV/pH unit. The electric potential difference between the pH-sensor and the Ag/AgCl reference electrode was amplified with a high impedance electrometer (VAJ-51, VEB RFT Mess-Elektronik, Germany) and displayed on a computer via a PCI-6024E AD converter.

### Localized illumination

The local illumination (local light, LL) was applied through a quartz optic fiber to cell regions located at a 1 or 1.5 mm distance from the point of measurements. The optic fiber with a diameter of 400 µm was connected to a source of white light (a light-emitting diode Luxeon LXK2-PWN2-S00, Lumileds, USA). The photon flux density (PFD) at the output of the light guide equaled to 500 µmol m^−2^ s^−1^; the length of LL pulses was 30 s. The free end of the light guide was fixed in a holder of a mechanical KM-1 micromanipulator (Chernogolovka, Russia) under the angle of 30–45° to a horizontal plane. After adjusting the light guide position in the view field near the cell, the optic fiber was displaced with a micrometric screw to a distance of 1–1.5 mm upstream the cytoplasmic flow with respect to the analyzed region.

During these measurement the whole cell was continuously exposed to blue background light (λ < 580 nm, 9 µmol quanta m^−2^ s^−1^ at the upper cell level). At low irradiance (8–10 µmol quanta m^−2^ s^−1^) long-distance communications between chloroplasts, manifested as *F’* changes in response to a bright pulse of local light, are pronounced to the highest extent.^16^ Low intensity background light is needed to maintain light-dependent photosynthetic enzymes in the active state. On the other hand, at high intensity of background light, the analyzed *F'* transients are masked by strong fluorescence quenching.^27^

### Microperforation

Microperforation of the cell wall was accomplished using glass microneedles with a tip size of ∼1 µm; the pipettes were pulled from Pyrex glass capillaries having an outer diameter of 1.1 mm. The experimental setup allowed independent positioning of the pH microelectrode, the micropipette for cell wall perforation, and a supporting glass capillary to prevent cell displacement by a stimulating needle. Perforation was performed as a short (for 1–2 s) shallow impalement followed by withdrawal of the pipette. Wortmannin (Enzo Life Sciences, Lausen, Switzerland) and cytochalasin D (Sigma, St. Louis, USA) were added from stock solutions in DMSO. This solvent at a concentration of 1–2% had no influence on cytoplasmic streaming and the membrane potential of characean cells.^19,28^

Figures represent the results of representative experiments performed in at least 4 replicates with different cells. Traces in figures are either individual or averaged curves calculated from several records, with *n* indicating the number of replicate measurements. Error bars on the kinetic curves represent standard errors of the means for measurements made in different cell locations. Error bars on longitudinal profiles of pH and PSII efficiency are standard errors determined from 2 replicate measurements of the profiles with 3 assays per point.

### Fluorescence pH imaging

*Chara* internodal cells were placed into artificial pond water supplemented with 10 µM fluorescein isothiocyanate (FITC) coupled to dextran with a molecular weight of 70000 Da (FITC–dextran 70 kDa; Sigma–Aldrich, St. Louis, MO, USA). The fluorescence of FITC is pH dependent and increases with alkalinity (e.g., ref. 29). Images were taken with a Leica (Mannheim, Germany) TCS SP5 confocal scanning microscope (excitation 488 nm, detection between 505 and 550 nm) or with a Zeiss (Jena, Germany) Axiovert 135 fluorescence microscope equipped with blue excitation filter cube (excitation range 450–490 nm, detection range 515–565 nm) and an AxioVision b/w camera. Images shown in this work are single images taken with a 4 × objective (numerical aperture 0.1) or with a 10 × objective (numerical aperture 0.3).
